# Nanoscale Phenotypic Textures of *Yersinia pestis* Across Environmentally-Relevant Matrices

**DOI:** 10.3390/microorganisms8020160

**Published:** 2020-01-23

**Authors:** Kanwal M. Iqbal, Massimo F. Bertino, Muhammed R. Shah, Christopher J. Ehrhardt, Vamsi K. Yadavalli

**Affiliations:** 1 H.E.J. Research Institute, University of Karachi, Pakistan 75270; kanwalmuhammadiqbal43@gmail.com (K.M.I.); raza.shah@iccs.edu (M.R.S.); 2 Department of Physics, Virginia Commonwealth University, Richmond, VA 23284, USA; mfbertino@vcu.edu; 3 Department of Forensic Science, Virginia Commonwealth University, Richmond, VA 23284, USA; cehrhardt@vcu.edu; 4 Department of Chemical and Life Science Engineering, Virginia Commonwealth University, Richmond, VA 23284, USA

**Keywords:** *Yersinia pestis*, atomic force microscopy, nanoscale, soil culture, 3D printed culture chamber

## Abstract

The persistence of bacterial pathogens within environmental matrices plays an important role in the epidemiology of diseases, as well as impacts biosurveillance strategies. However, the adaptation potentials, mechanisms for survival, and ecological interactions of pathogenic bacteria such as *Yersinia pestis* are largely uncharacterized owing to the difficulty of profiling their phenotypic signatures. In this report, we describe studies on *Y. pestis* organisms cultured within soil matrices, which are among the most important reservoirs for their propagation. Morphological (nanoscale) and phenotypic analysis are presented at the single cell level conducted using Atomic Force Microscopy (AFM), coupled with biochemical profiles of bulk populations using Fatty Acid Methyl Ester Profiling (FAME). These studies are facilitated by a novel, customizable, 3D printed diffusion chamber that allows for control of the external environment and easy harvesting of cells. The results show that incubation within soil matrices lead to reduction of cell size and an increase in surface hydrophobicity. FAME profiles indicate shifts in unsaturated fatty acid compositions, while other fatty acid components of the phospholipid membrane or surface lipids remained consistent across culturing conditions, suggesting that phenotypic shifts may be driven by non-lipid components of *Y. pestis*.

## 1. Introduction

Several non-spore-forming bacterial pathogens can persist in environmental matrices outside of transmission vectors or mammalian hosts for extended periods of time. Examples include *Brucella abortus* (agent of brucellosis) [1,2], *Pseudogymnoascus destructans* (agent of white nose syndrome) [3], *Burkholderia pseudomallei* (agent of melioidosis) [4], and *Yersinia pestis* (agent of plague) [5]. For *Y. pestis* in particular, survival and persistence in matrices such as soil has been hypothesized to contribute to infections of susceptible hosts [6], and the periodicity of plague epidemics in many parts of the world. A number of field observations and laboratory studies have demonstrated survival of *Y. pestis* in soil for periods ranging between days to months [7,8]. More recent case studies have shown not only viability of *Y. pestis* after several weeks in desert soil but also that organisms are able to maintain virulence [5]. However, the mechanisms of long-term persistence within soil matrices without arthropod or mammal hosts remains poorly understood. For other gram negative pathogens such as *E. coli* and *B. pseudomallei*, biochemical changes to the cell membrane and/or ultrastructure of the cell has been associated with soil-mediated survival [9,10], but this has not been investigated explicitly in *Y. pestis*.

One of the obstacles to studying bacterial phenotypes in situ comes from the difficulty in mimicking the complex range of environmental factors within the laboratory, both abiotic (e.g., pH, O_2_, mineral composition) and biotic (e.g., presence of soil microbiota, nutritional regime). As such, a number of experimental strategies have been developed. These include growth medium formulations that contain solubilized organic matter extracted directly from soil [11], as well as direct inoculation and recovery of target organisms within soil matrices. The latter strategy tends to be limited by low recovery efficiencies and/or damage to the cells [12]. Owing to this, many studies of *Y. pestis* persistence in soil have focused on culture-based viability assays or genetic analyses [7,13]. One promising, but unexplored strategy for studying environmental persistence of *Y. pestis* is the use of diffusion chambers that contain target organisms within a small volume/area, and expose them to compounds from the external environment while preventing contamination from in situ bacterial communities. Diffusion chambers have been used to study uncultivable bacteria directly within environments such as soil, marine systems, and river water [14,15,16,17]. However, to date, such growth chambers have also not been used for studying phenotypes and biochemical signatures of human pathogens following long term persistence in soil.

In this work, we report on the nanoscale phenotypic textures of *Y. pestis* across environmentally-relevant matrices. This study is enabled by the design and fabrication of a novel diffusion chamber for studying *Y. pestis* under controlled microenvironments, specifically soil. Organisms within the chamber can be inoculated onto agar surfaces and exposed to a bi-directional flow of nutrients and other (bio) chemical compounds between the soil matrix and the chamber. Importantly, the chamber prevents migration of bacteria either into or out of the chamber, thereby isolating the bacteria to specific environmental effectors without contamination from other microbial communities. The chamber is designed such that it can be 3D printed in an “on-demand” fashion to suit experimental replicates or field-based studies. To demonstrate potential utility for phenotypic characterizations, the chamber was used to incubate cells within a soil matrix. The cells collected could then be profiled at both the population (ensemble) level and at the single cell level. At the population level, we focused specially on membrane fatty acid composition since the persistence of many bacterial pathogens in environmental matrices is often facilitated through compositional and structural shifts in the cell membrane [18,19,20,21]. At the single cell level, we characterized the micro- to nanoscale size, morphology, and significantly, the hydrophobic properties of the cell surface. Hydrophobicity measurements were used to determine the phenotypic characteristics of *Y. pestis*, following incubation within soil. Techniques to analyze the hydrophobicity of cells at the *single* cell level have not often been used for such studies, particularly for pathogens such as *Y. pestis*. As we show, the presence of soil causes differences in the size of the bacteria, as well as the hydrophobicity of the cell surface. Such techniques can therefore lay the foundation to better elucidate the long-term effect of soil and specific microenvironments on the survival and persistence of bacterial pathogens.

## 2. Materials and Methods

### 2.1. Chemicals and Instruments

We purchased 1-undecanethiol from Sigma-Aldrich (St. Louis, MO, USA). 3 aminopropyltri-ethoxysilane (APTES), glutaraldehyde (25% aq. solution), Phosphate-buffered saline (PBS pH 7.4) (11.9 mM phosphates, 137 mM sodium chloride and 2.7 mM potassium chloride) and ethanol (200 proof or 100% ethanol) were purchased from Fisher Scientific (Waltham, MA USA). Mica was purchased from Ted Pella (Redding, CA, USA). AC240TS cantilevers (Olympus, Center Valley, PA, USA) were used for non-contact mode imaging in air, while gold coated TR800PB cantilevers (Olympus) were used for force measurements. To prevent contamination, cantilevers were cleaned using an UV/Ozone Procleaner (BioForce Nanosciences Inc. Ames, IA, USA) before use. Imaging and force spectroscopy experiments were performed on an Asylum Research MFP-3D atomic force microscope (Oxford Instruments, Santa Barbara, CA, USA).

### 2.2. Fabrication of 3D Printed Cell-Culture Chamber

**Figure 1:**
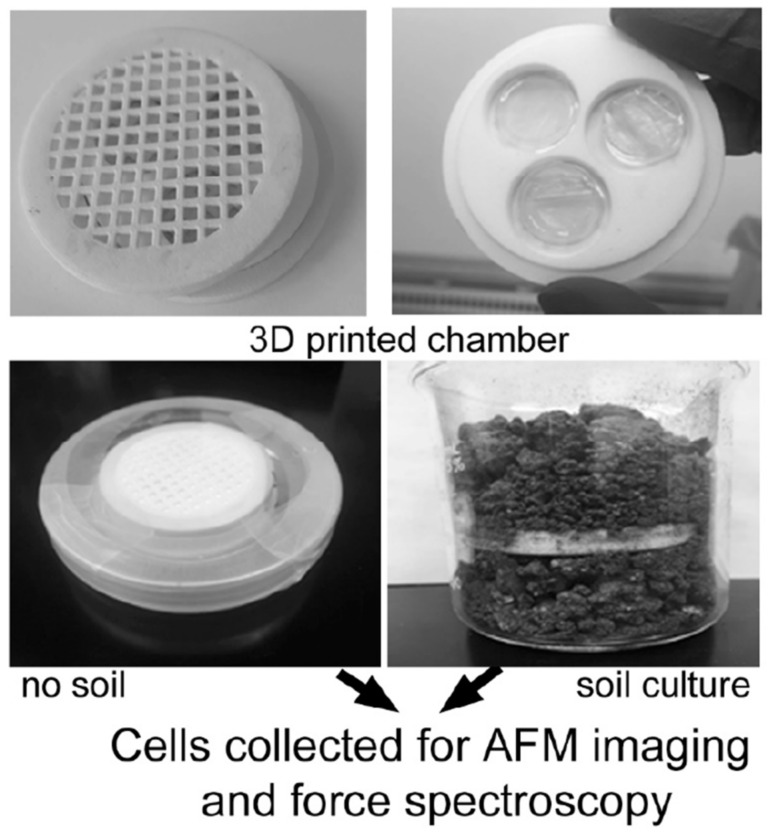
The 3D printed cell culture incubation chamber used to investigate culture of *Yersinia pestis* under controlled environments. After streaking cells onto each of three separate agar plugs within chamber, parafilm is used to seal and chamber is placed into either no soil or soil conditions, and allowed to grow for 48 h at 30 °C.

Resin chambers were printed using a Form 2 resin printer (Formlabs Inc., Somerville, MA, USA). The chamber is designed to hold three small agar plugs (40 mm diameter) as the substrate for bacterial inoculation (Figure 1). The chamber was configured and fabricated as previously reported by our group [22]. Briefly, the agar plugs are held in place by two identical lid pieces with gridded meshes. After printing, the chamber pieces were autoclaved and allowed to cool before assembly. The mesh pieces are interchangeable and allow adequate space for nutrient flow to the bacteria inside the chamber. The inner portion fits snugly inside the meshes. The chamber size was optimized to precisely match the dimensions of the filters. The grid mesh comprising the top and bottom pieces of the incubation chamber were made of the same material as the interior piece holding the agar plugs (polyamide nylon plastic). The pores within the chamber mesh are 4 mm^2^. The filters situated inside the chamber are polyethersulfone (PES) with a pore size of 0.22 µm. A silicone sealant was used to attach a 47 mm diameter, 0.03 µm polyethersulfone (PES) membrane filter (Sterlitech Corporation, Kent, WA, USA) to the outer mesh components. Next, three 40 mm agar plugs were placed inside the inner chamber piece. After inoculating bacteria onto the plugs, the lid pieces were placed above/below the plug assembly. The outer edge was then wrapped in parafilm before incubation within soil (controlled microenvironment) (Figure 1). The pore size of the filter prevents movement of bacteria in or out of the chamber as well as for isolated bacterial analysis.

### 2.3. Cell Culture

An avirulent/Biosafety Level 2 strain of *Y. pestis* was obtained through BEI Resources, NIAID, NIH (Manassas, VA, USA): Strain KIM Derivative 19 (D19), NR-4681. This was used for all experiments. For starter cultures, a single colony of *Y. pestis* KIM from Trypticase Soy Agar (TSA) plate was inoculated into 2 mL of TSB (Trypticase Soy Broth (Millipore Sigma #105459)) and grown overnight at 30 °C, 225 rpm. Two mL of this culture was added to 100 mL TSB and grown overnight at 30 °C, 225 rpm. The overnight cell suspension was then washed three times in sterile 1x PBS buffer and the final cell pellet re-suspended in 10 mL 1x PBS. Next, 200 µL of the cell suspension was plated onto either Trypticase Soy Agar (TSA) or agar substrates with no other source of carbon/nitrogen compounds (‘agar’). In each case, after inoculation, cultures were grown at 30 °C for 48 h. in a stationary incubator. Following 48 h. growth, cells were harvested from plates using sterile cell scrapers and inoculation loops and inactivated with 100% MeOH, washed twice, then re-suspended in deionized water until further analysis [23]. Growth curves for *Y. pestis* KIM were generated in Tryptic Soy Broth (TSB) as well as four different SESOM formulations (i.e., liquid broths containing only soil-derived organic matter) which vary in organic carbon content, ranging between ~3% and ~10%. All growth curves were collected over the course of ~60 h (Figure S4).

### 2.4. Chamber-Grown Bacteria

For experiments involving the diffusion chamber, a small inoculum of *Y. pestis* (~10 µL) was streaked onto TSA plates and allowed to grow for 48 h at 30 °C. Biomass was then harvested using sterile inoculation loops and transferred to TSA plugs placed inside the incubation chambers. When agar-only plugs were used inside chambers, bacteria were harvested from TSA plate and washed three times in sterile 1x PBS, then transferred to agar plugs with sterile inoculation loops. After bacterial inoculation, growth conditions for all chambers were ~48 h at 30 °C, inside a stationary incubator. As control experiments, bacteria were grown in the chambers not exposed to soil. This experiment was used to verify that the chambers themselves were not affecting cell behavior, independently of the soil. Chambers were placed inside petri dishes on top of a ~0.4 cm tall platform, raising the chamber above ~3.5 mL water added to the petri dish to retain humidity. Two layers of parafilm were applied to seal the petri dish.

In order to confirm that the chamber apparatus prevented in situ soil microorganisms from colonizing the agar plugs while still permitting diffusion of chemical components, un-inoculated plugs were sealed inside a series of chambers that were incubated directly in a soil matrix for at least three days. After the incubation period, the plugs were removed from the chamber, and vortexed directly with 5 mL of Tryptic Soy Broth (TSB). The entire broth solution was then plated onto five Tryptic Soy Agar (TSA) plates (1 mL each) and incubated for 72 h at 30 °C. No colonies were observed on TSA plates. Viability of organisms before and after incubation in the chamber was initially assessed through plate counts on TSA. Viability of cells following growth on Tryptic Soy Agar (TSA) and SESOM-agar formulations was also assessed using a ‘Live/Dead’ assay (Data shown in Figure S5). Starting cell suspensions were created by harvesting one loopful (~10 µL) of bacterial colonies from an overnight TSA culture and mixing with 5 mL of 1x PBS. Next, 50 µL of the starting cell suspension was spread onto each agar plug. After incubation, biomass was removed from each agar plug using an inoculating loop and eluted in 5 mL of 1x PBS. Serial dilutions of this cell suspension as well as the starter cell suspension were then inoculated onto TSA plates (20 µL each). Colonies were counted after incubation for 48 h at 30 °C. No distinct differences were observed in the number of viable cells between agar-only controls, agar plugs within the chamber with no surrounding soil, and agar plugs within the chamber that was exposed to soil (Table S1), indicating that the chamber itself did not negatively affect organisms’ viability, nor did the presence of soil matrix. However, variability was observed between replicate agar plugs within the same chamber, which may be due in part to spatial heterogeneity of soil matrix with respect to the chamber.

### 2.5. Culturing in SESOM Medium

Soil extracted solubilized organic matter’ (SESOM) solution using dried soil were prepared as per a prior protocol [11]. The extracted SESOM broth made from a mixture of 50 g top soil and 250 mL deionized water. The starting soil consisted of ~40 g of the A horizon (i.e., top 10 cm). The SESOM extract broth was purified through a 0.2 µm filter, combined with agar for a final concentration of 1.5% by weight agar, and then autoclaved.

### 2.6. Chamber Experiment with Soil

Chambers exposed to soil were placed inside a 500 mL beaker and completely covered with soil to which non-sterile tap water was added to achieve a water content of ~50%. The amount of water added was varied due to the initial moisture content of soil obtained. Two layers of parafilm were applied to the top of beaker to retain humidity. Organic matter content was determined by loss-on-ignition (LOI) method following the Environmental Protection Agency protocol NCEA-C-1282 [24]. About 25 g of each dry soil sample was placed in a ceramic crucible which was then heated to 360 °C overnight. The sample was then cooled in a desiccator and weighed. The percentage of organic matter content was calculated as the difference between the original and final sample weights divided by the initial sample weight. Organic matter was estimated as 14.6% of the soil used for chamber incubation experiments.

### 2.7. Imaging and Atomic Force Microscopy

All cells were inactivated using previously reported protocols using methanol, that was earlier shown by our group to not affect the cell surfaces [23]. This ensured that the cells were completely inactivated prior to imaging which was conducted outside of a biological safety cabinet. For force spectroscopy, cells were chemically immobilized using APTES and glutaraldehyde. Mica slides were incubated in APTES vapor in a vacuum desiccator for 16 h, then immersed in 1 mL 2% (*v*/*v*) glutaraldehyde-water solution for 1 h and finally rinsed with deionized water. 30 μL of cell suspension in water was pipetted out on modified mica surfaces. AC 240TS cantilevers (spring constant ~2 N/m, resonance frequency 70 kHz) were used for imaging in air and characterization of the surfaces in non-contact mode. Force spectroscopy experiments were carried out using a gold coated cantilever (TR800PB spring constant ~0.167 N/m, resonance frequency ~50 kHz). In order to observe the surface hydrophobicity, gold-coated cantilevers were functionalized with 1-undecanethiol which can bind hydrophobic moieties on the cell surface (hydrophobic interaction). Care was taken to use freshly cleaned tips for each experiments to prevent contamination [25]. At least 400 force curves were obtained at specific trigger for each sample, allowing binding with 0.99 s contact time and then retracting. A “force mapping” technique was used to visualize the hydrophobicity of the cell surface. These data were represented in the form of area maps as 20 × 20 curve arrays with a spacing of ~25 nm each resulting a representative scan of 0.5 µm^2^ (500 nm × 500 nm) of the cell surface.

### 2.8. Fatty Acid Profiling

Fatty acid profiles for bacterial samples were generated using the ‘Instant FAME’ Method following manufacturer’s instructions (MIDI Inc. Newark, DE, USA). A set of three reagents are used to extract fatty acids, derivatize to methyl esters, and separate into an organic phase prior to Gas Chromatography (GC) analysis. First, 10 mg of wet biomass was removed from the agar substrate and placed in 2 mL glass tube. Cell lysis and derivatization of fatty acids was performed by adding 250 µL of 5% potassium hydroxide, 95% methanol solution (MIDI Inc. P/N 7020-101, Newark, DE, USA). The mixture was then pulse mixed for 10 s at 3000 rpm. Next, 125 µL of analytical grade hexanes was added, followed by pulse mixing as before for 10 s. This step partitions the fatty acid methyl esters into the organic phase. Finally, 250 µL of dyed aqueous hydrochloric acid solution (MIDI Inc., P/N 7020-303) was added to the mixture to help visualize the phase boundary and facilitate transfer of the hexane layer to a new glass vial (~100 µL).

FAMEs were then analyzed with an Agilent 7890A gas chromatograph equipped with an HP-Ultra2 column (Agilent Technologies, P/N 19091B-102E) and hydrogen as the carrier gas (1.4 mL/min flow rate and 21.2 psi). The split ratio was set to 20:1. The oven temperature program started at 170 °C and increased to 288 °C at 0.5 °C/s and then increased from 288 °C to 310 °C at 1 °C/s. Identification of FAME structures and quantification of relative abundance was accomplished using the MIDI Microbial Identification Sherlock software and calibration standards (MIDI Inc., P/N 1300-C) according to the manufacturer’s protocol.

## 3. Results and Discussion

### 3.1. Chamber Design and Cell Culture

Understanding the chemical and physical properties of *Y. pestis* cells that occur with survival in soil matrices is critical for elucidating uncharacterized aspects of the transmission life-cycles of plague. Towards this objective, we designed a customizable and cost-effective device to expose target organisms to the complex abiotic and biotic components of soil matrix without (a) the risk of contamination from endemic soil bacteria, or (b) the difficulty in directly harvesting target bacteria for phenotypic analysis. In order to grow *Y. pestis* in a soil environment, custom-engineered 3-D printed growth chambers were prepared (Figure 1) [22]. Initially, it is useful to compare these devices with previously reported chambers designed to expose cells to external environments. Prior devices have generally been optimized for capture and/or manipulation of single bacterial cells through a series of mini-chambers (often 1 cell/well). Most of these systems are designed primarily for high throughput analysis and manipulation of single cells for targeting unculturable bacteria or rapid screening of antimicrobial compounds [26,27]. However, for phenotypic profiling, a device and workflow are needed that can be used to inoculate and then collect a greater number of target organisms from the soil matrix. Moreover, this enables the ensemble behavior of cultures which is representative of the realistic environment, which has not been thoroughly studied.

In initial experiments, two conventional growth media were used: agar substrates with no other exogenous source of carbon or nitrogen compounds, and Trypticase Soy Agar (TSA) which contains enzymatic digests of soy and casein. The two media were then used as inoculation substrates within 3D printed growth chambers. The first set of experiments were designed as controls to investigate the impact that the chambers themselves (in the absence of soil) had on cell properties. Analysis was conducted on cellular morphology and cell surfaces as discussed further below. As a second control, cells were cultured on agar containing SESOM extract, which should be similar (although not identical) to the biochemical and abiotic environment of the chamber when incubated in soil.

### 3.2. AFM Imaging of Single Y. pestis Cells

To visualize the cells at a single cell level and study their nanoscale surface textures, the technique of atomic force microscopy (AFM) was used. The AFM is a tool that has been widely used over the past couple of decades in biological research to visualize cells with nanoscale resolution, characterize cell surfaces, as well as to measure physicochemical properties and force interactions [28,29,30]. Nanoscale images of *Y. pestis* using scanning electron microscopy (SEM) have been reported at different growth temperatures, revealing capsular antigens as a granular layer on the surface [18]. *Y. pestis* surface morphology was studied with or without glucose in growth media [31], and with or without the hemin storage gene [32]. However, to date, *Y. pestis* has not been well studied at the nanoscale. Our group earlier presented some of the first reports using nanoscale imaging and biochemical mapping at the single cell level, for *Y. pestis* cultured across standard laboratory media and across different temperatures [23,33].

**Figure 2:**
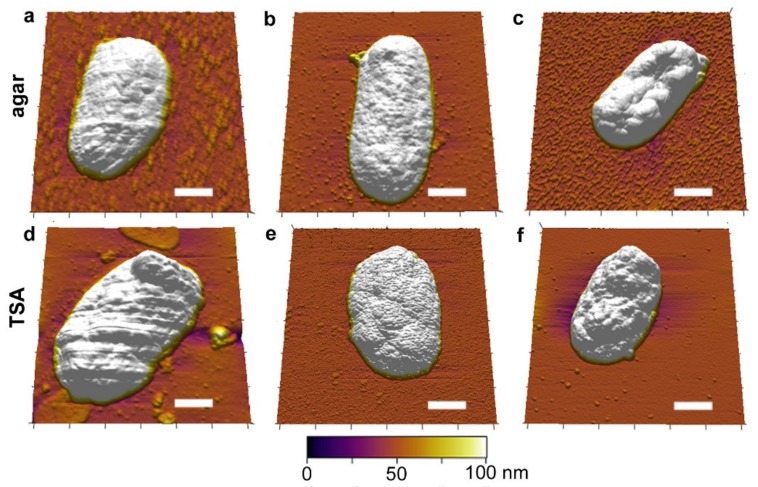
3D images of *Yersinia pestis* cells obtained using atomic force microscopy (AFM) imaging. Cells were cultured under different conditions—top row in agar and lower row in TSA. (**a**) agar (plate), (**b**) agar (in 3D printed chamber with no soil) (**c**) agar (3D printed chamber with soil) (**d**) TSA (plate) (**e**) TSA (in 3D printed chamber with no soil) (**f**) TSA (3D printed chamber with soil). Scale bar on all images = 500 nm. Note that representative images are presented and may not correspond to the statistical data shown below, which were collected for several cells. The presence of some debris is noted on the samples cultured on the plates in both agar and TSA.

**Figure 3:**
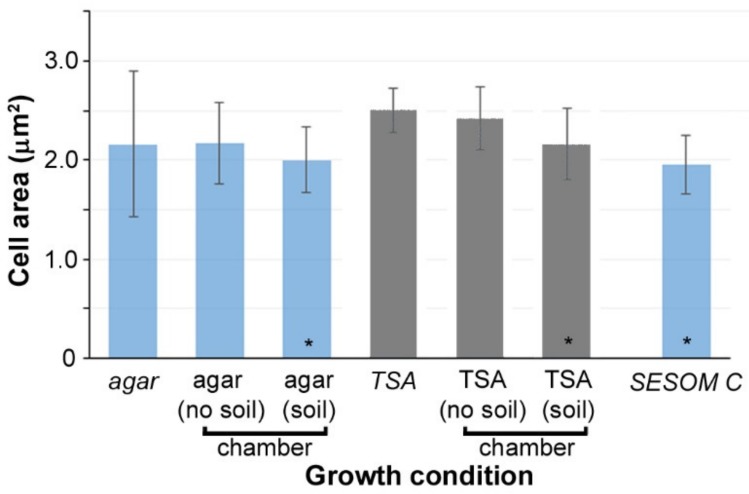
The mean cell size for *Yersinia pestis* grown under different conditions. Conditions in *italics* represent the growth in regular plates, whereas the others were grown in the 3D printed cell culture chambers. *—represents the soil or soil-like conditions (SESOM). In general, the cell size did not change whether grown on plates or in the chamber, reflecting that the chamber itself did not influence cell size. However, in both soil and soil-like conditions, the average cell size decreased. At least five cells were analyzed for each condition.

High resolution nanoscale images were obtained for the *Y. pestis* cells under different conditions (Figure 2). Raw data is shown in Figures S6 and S7. Cells grown in agar and TSA plates, and in the chamber (with and without soil) were compared (5–10 cells were considered for each condition for statistical analysis and data are presented as mean ± s.d.). Colonies were counted after incubation for 48 h at 30 °C. Specifically, the sizes of the cells are considered on plates and in the chamber in the absence and presence of soil. Cells grown on agar plates (2.16 ± 0.73 µm^2^) and in the chamber (2.17 ± 0.41 µm^2^) as well as the cells grown on TSA plates (2.50 ± 0.22 µm^2^) and in the chamber (2.42 ± 0.32 µm^2^) could not be differentiated based on size (Figure 3). These data indicate that the chamber itself does not cause any adverse impact on the cell growth. Interestingly, the cells grown in the soil were observed to be smaller in comparison to their counterparts not grown in soil. For agar + soil, the cells were 2.00 ± 0.33 µm^2^, whereas for TSA + soil the cells were 2.16 ± 0.36 µm^2^. This decrease (~10%) suggests that incubation within soil matrix with or without TSA may yield cell sizes comparable to those observed when cultivated on agar base medium.

As a control, *Y. pestis* cells grown in SESOM media were also smaller in size (1.98 ± 0.15 µm^2^) showing a consistency in this trend (Figure S1). The cells did not display any specific differences in terms of their shape or their surface roughness across different conditions, including SESOM agar (Figure S2). There was also no observable difference in the aspect ratio of the cells. It is important to note that in Figure 2, we have shown representative images of the cells reflective of their overall diversity. However, we did observe a decrease in average cell size when cells were cultivated in the presence of soil or on SESOM agar compared to cells cultured on TSA or agar (Figure 3). Reductions in cell volume are commonly observed in bacteria when exposed to oligotrophic environments [34,35,36,37]. The fact that average cell size decreased when *Y. pestis* was grown on nutrient rich agar substrate but in the presence of soil may indicate that the presence of soluble soil components diffusing through the chamber may affect phenotype of *Y. pestis*.

### 3.3. Cell Surface Hydrophobicity Using Force Spectroscopy

Cell surface hydrophobicity (CSH) plays an important role for the attachment or detachment of bacteria from solid surfaces, formation of biofilms, removing contaminants from soil and water, and, importantly, can affect infectivity/virulence [38]. Therefore, a quantitative analysis of bacterial cell-surface hydrophobicity has the potential to provide information about interactions between bacteria, and between bacteria and surfaces that can lead to long term persistence. In this work, hydrophobicity measurements were used to determine the phenotypic characteristics of *Y. pestis*, following incubation within soil. There have been various studies to determine CSH, mostly at the bulk or ensemble level. A summary of some relevant experiments is presented in Table 1. To date however, techniques to analyze the hydrophobicity of cells at the single cell level have not often been used for such studies, particularly for pathogens such as *Y. pestis*. As part of this study, we explored how the soil microenvironment affects the morphology and the biochemical properties of the cell surface, specifically its hydrophobicity at the single cell level. Following the nanoscale imaging discussed above, we developed a strategy to probe the distribution of hydrophobic residues for individual *Y. pestis* cells.

Force spectroscopy analysis at the single cell level demonstrates how the external microenvironment plays roles in modulating both the morphology and key components of the bacterial surface. The AFM not only measures the interaction forces between ligands and receptors, but can also spatially map sites on the cell surface. The interactions between a functionalized AFM tip and the sample (e.g., cell surface) are measured by analyzing the force-distance curves obtained in a raster scan of an area of the surface [49,50]. In this study, the effect of soil microenvironments on the cell surface hydrophobicity was probed by using AFM cantilevers that were functionalized with pendant hydrophobic (–CH_3_) groups. *Y. pestis* cells were obtained as discussed above, and cultured on agar and TSA plugs in the culture chamber in the presence and absence of soil. As a control, cells grown on agar plates (no chamber) were used. Initially, all cells were probed with un-functionalized cantilevers to demonstrate that the tip–surface interactions observed were indeed the hydrophobic interactions and not non-specific interactions. As noted in earlier works, the unmodified cantilevers are inert gold surfaces and the force spectroscopy represents the hydrophilic and non-specific interactions on the cell surface [51,52]. Data showing force maps for un-functionalized cantilevers are presented in the Supporting Information (Figure S3).

**Figure 4:**
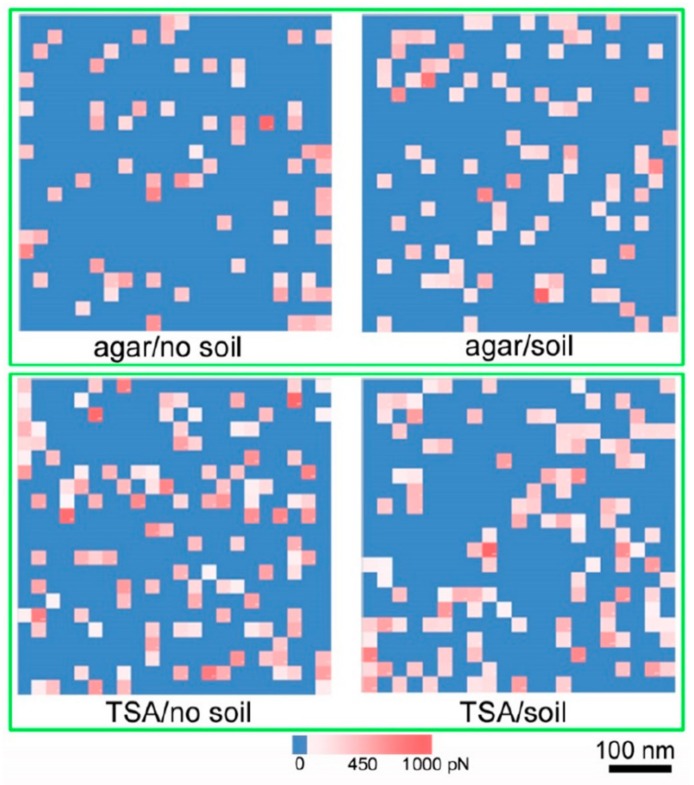
Surface hydrophobicity maps of *Yersinia pestis* cell surfaces grown under different conditions in the 3D printed culture chamber. The cell surfaces were probed with a hydrophobic AFM tip. The maps are color coded as blue = no interaction with tip, white to red = increasing interaction force with tip (ranging from 200 pN to 1 nN). The points are ~25 nm apart, thereby each square presents a profile of a 0.5 µm^2^ area of the cell (scale bar = 100 nm). The number of non-blue areas are representative of the points of interaction (hydrophobic groups) on the cell surface.

**Figure 5:**
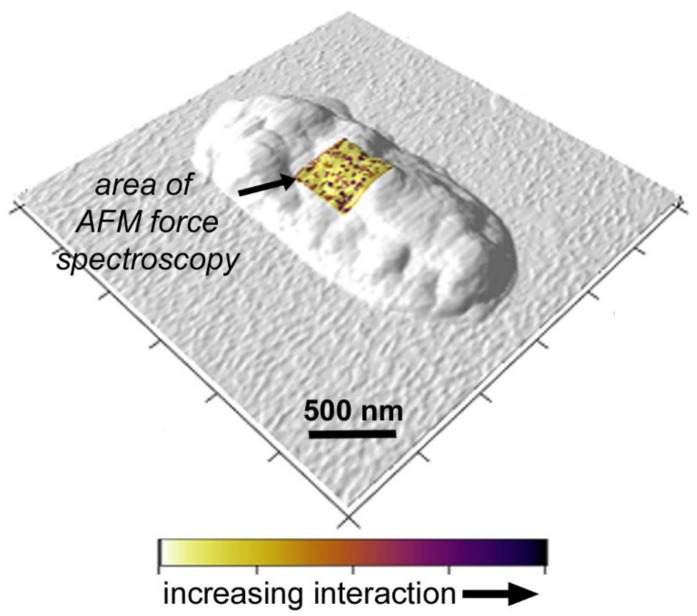
Image showing the force mapping of the *Yersinia pestis* cell surfaces. Here, the colors of blue-white-red used in Figure 4 are presented as yellow to purple to better show how the surface hydrophobicity varies across the cell surface (dark purple = higher interactions (points of hydrophobicity), white to yellow = no interaction). A 500 × 500 nm^2^ area of the cell is interrogated.

The interaction data are presented as a force map in Figure 4. Each surface presents 20 × 20 force curves taken ~25 nm apart resulting in a visualization of a 500 nm × 500 nm portion of the cell surface (0.5 µm^2^). In the image, the blue regions represent “no-interaction” with the functionalized cantilever. The color scale of white to red represent increasing interaction force (binding with the tip, ranging from 150 pN to 1 nN). The range of force is line with that recorded for hydrophobic interactions measured via single molecule force spectroscopy [53]. Therefore the % of points with non-blue color is representative of the hydrophobic groups on the cell surface. As observed in the figure, the hydrophobicity of the cell surfaces was higher on TSA grown cells in comparison to cells grown on agar, irrespective of whether they were cultured in soil or not. Interaction data is shown in Table S2. For instance, agar (no-soil) had 13.8% interactions in comparison to TSA (no-soil) at 24.4%. In comparison agar (soil) had 17.8% interactions in comparison to TSA (soil) at 26.3%. This “force map” can be visualized on the cell surface by the overlay presented in Figure 5. This image shows how the surface hydrophobicity correlated with the underlying cell surface. Note that the color scale used in this figure was chosen to enhance clarity, but essentially follows the same gradient used in Figure 4. *Y. pestis* cells have amphipathic lipopolysaccharide molecules consisting of lipids conjugated to core polysaccharide residues and an O-antigen molecule. The variation of sugar residues in LPS core OS can alter the distribution between the hydrophilic and hydrophobic portions of the molecule, resulting in a change in the surface hydrophobicity of the whole cell [54,55]. Additionally, variation in nutrient content of growth medium can affect the presence and concentration of hydrophobic peptides on the cell surface [56]. Previous studies using *Pseudomonas aeruginosa* [57] and *Y. enterocolitica* [58] found that surface hydrophobicity is more pronounced in enriched media.

Results from this study indicate that cultivation within soil matrix may similarly influence hydrophobicity of the *Y. pestis* cell surfaces. For cells harvested from agar substrates hydrophobicity increased from 13.8% to 17.8%. For cells harvested from TSA, hydrophobicity showed a slight increase from 24.4% to 26.3% when incubated in the presence of soil matrix. This suggests a physical/chemical change in soil microenvironments that includes a decrease in cell size with a concomitant increase in surface hydrophobicity. Earlier studies at the bulk level have shown a correlation between CSH and the adhesion of bacteria to soil particles. This appears to be the case even for pathogenic bacteria such as *Y. pestis* in soil environments. It is important to note the control in which the force map was taken of cells cultured on agar plates (no chamber—14.3%) showed similar interaction behavior in comparison to cells cultured on agar (chamber—13.8%). This further confirmed that the chamber itself is not affecting the cellular dynamics.

### 3.4. Whole Cell Fatty Acid Profiles

Various bacteria have developed mechanisms to alter their cell membrane composition in order to maintain fluidity and functionality in response to external environmental conditions such as soil. It is known that the phenotypic adaptions of cell membrane structures can be modulated by the degree of unsaturation, the chain length, branching or cyclization of bacterial membrane fatty acids [59,60]. However, these adaptations in soil remain poorly understood. To complement single cell analyses, whole cell fatty acid profiles of *Y. pestis* were analyzed. As seen in Table 2, the relative abundance of key membrane FAME biomarkers: 14:0-3OH; 16:1 ω7c, 16:0, 18:1 ω7c, 18:0, and 19:0 cyclo ω8c show some similarities across diffusion chamber cultures incubated with and without a surrounding soil matrix and using both TSA and base agar substrates. The conserved nature of the overall fatty acid profile across soil and non-soil conditions is not unexpected given that previous studies have shown that membrane composition in *Y. pestis* is largely influenced by growth phase, temperature, and nutrient availability [61,62].

For some gram negative bacteria, other abiotic factors can induce shifts in abundance of certain fatty acid biomarkers within the membrane (e.g., pH [63,64] temperature [65] or exogenous organic compounds [66]). The absence of similar shifts in this study therefore indicates that the particular soil type used for chamber incubations did not significantly alter the abiotic environment or growth phase dynamics of *Y. pestis*. Interestingly, there was a small variance (~10% increase) as noted by a higher proportion of unsaturated fatty acids (16:1 and 18:1) in the presence of soil for both TSA and agar cultures. This suggests a possible up-regulation of the biosynthetic pathway in the presence of soil [60], or a difference in the growth phase across soil and non-soil cultures at the time of cell harvesting [61]. Of note within the fatty acid profiles is the consistency of 14:0 3-OH abundance since this is considered a functional indicator of the outer LPS layer rather than the phospholipid membrane [55]. It must be noted that surface probes such as AFM reflect the hydrophobicity of the exterior of the cell membrane whereas the FAME biomarkers reflect the overall composition. It is therefore difficult to point to a direct correspondence between specific biomarkers from these techniques. Indeed, surface hydrophobicity differences observed with single cell analyses could also be derived from non-lipid components of the *Y. pestis* cell surface. However, taken together, such studies (including the use of devices that allow for precise control of conditions) have the power to provide unique insights into the nanoscale (morphological) and biochemical (e.g., membrane phospholipid fatty acids) adaptation strategies to various external environments.

## 4. Conclusions

In summary, the goal of this study was to demonstrate a new approach to characterizing phenotypic signatures in bacterial pathogens in environmental matrices. Using a 3D printed diffusion chamber, *Y. pestis* KIM organisms were characterized after incubation directly both with and without a surrounding soil matrix. Using such chambers, it is also possible to map the spatial and temporal dynamics of “microbial hot spots”. Results obtained using both nanoscale and ensemble techniques suggest that phenotypic shifts are associated with presence of soil matrix including decrease in cell size and an increase in surface hydrophobicity. Future studies will focus on understanding the adaptations of *Y. pestis* to specific environmental factors including the effects of temperature, and chemical and biological xenobiotics in the soil. This work can provide foundation for future studies that characterize *Y. pestis* survival strategies across range of environmental conditions and matrices in which *Y. pestis* has been observed to persist.

## Figures and Tables

**Table 1:** Effect of growth conditions on hydrophobicity.

Bacteria	Media	Technique	Comments	Ref
*Pseudomonas* sp., *arthrobacter* sp., *A. globiformis*, *E. coli*.	Mineral salt medium. Acetate, ethanol, mannitol, glucose and a-xylene as growth substrate	Water contact angle	Investigated the influence of substrate and growth conditions on hydrophobicity and electrophoretic mobility	[39]
*E. coli*, *P. putida*, *F. breve*, *S. marcescens*, *A. calcoaceticus*	LB	BATH, MAC, HIC	Determined the cell surface properties directly in waste water.	[40]
*L. monocytogenes*	TSYE and BHI	MATS	Hydrophobicity trend was TSYE > BHI	[41]
*S. epidermidis*	HBA, BHIA, BHIB, TSB and PPB	HAA	Hydrophobicity trend was HBA> BHIA > BHIB> TSB> PPB	[42]
*Lactobacillus* sp.,	De Man-Rogosa-Sharpe medium	Water contact angle, force spectroscopy	Determined the changes in cell surface hydrophobicity in response to ionic strength	[43]
*Mycobacterium bovis*	Sauton medium	Chemical force microscopy	Measured hydrophobic forces on cell surface	[44]
*E. coli*	LLB and SLB	Water contact angle	Characterized hydrophobic and hydrophilic parts of cell surface	[45]
*E. coli*, *S. aureus*, *A. niger*	LB and PSM	MATH	Determined DEP degradation using hydrophobicity.	[46]
*S. aureus*	TSB	MATH	Determined cell surface hydrophobicity increase with temperature.	[47]
*E. coli*	LB	Water contact angle	Determined the level of bacterial adhesion with hydrophobicity	[48]

Details- BATH = bacterial adhesion to hydrocarbons, BHI = brain heart infusion, BHIA = brain heart infusion agar, BHIB= brain heart infusion broth, DEP = diethyl phthalate, HAA = n-hexadecane adherence assay, HBA = horse blood agar, HIC = hydrophobic interaction chromatography, LB = luria broth, LLB = liquid luria bertani media, MAC = microsphere adhesion to cells, MATH = microbial adherence to n-hexadecane, MATS= Microbial adhesion to solvents, PPB = proteose peptone broth, PSM = potato sucrose medium, SLB = Solid luria bertani agar, TSB = tryptic soy broth, TYSE= trypticase soy broth supplemented with 6 g/L yeast extract.

**Table 2:** Fatty acid profiles of *Y. pestis* in Tryptic Soy Agar (TSA) and agar. The mean relative abundance of each biomarker is given with one standard deviation (*n* = 6 for each culturing condition).

	14:0 3-OH	16:1 ω7c	16:0	17:0 Cyclo	18:1 ω7c	19:0 Cyclo
TSA-no soil	1.3 ± 0.9	6.7 ± 4.4	39.0 ± 3.0	41.7 ± 4.7	5.2 ± 2.8	3.6 ± 1.0
TSA-soil	1.9 ± 1.5	8.1 ± 3.5	36.9 ± 1.7	41.0 ± 3.9	6.1 ± 2.4	3.9 ± 1.5
Agar-no soil	1.7 ± 0.2	3.4 ± 1.1	36.1 ± 0.6	45.7 ± 1.3	3.4 ± 1.6	7.4 ± 1.2
Agar-soil	1.5 ± 0.1	5.2 ± 2.0	34.8 ± 1.7	43.0 ± 2.3	5.7 ± 1.7	6.4 ± 0.6

## Supplementary Materials

The following are available online at https://www.mdpi.com/2076-2607/8/2/160/s1, High resolution AFM images, surface roughness data, force maps, growth curves, cell viability analysis are presented in the Supporting Information.
