# Supplementary material for: Nanoscale Phenotypic Textures of Yersinia pestis Across Environmentally-Relevant Matrices
Source: Microorganisms. 2020 Jan 23;8(2):160. doi: 10.3390/microorganisms8020160 (PMC7074701; doi:10.3390/microorganisms8020160)
Supplement: Supplementary file 1 [file microorganisms-08-00160-s001.pdf]

Supporting information for manuscript:

Nanoscale Phenotypic Textures of *Yersinia pestis* Across Environmentally-Relevant Matrices

Kanwal M. Iqbal [1], Massimo F. Bertino [2], Muhammed R. Shah [1], Christopher J. Ehrhardt [3] and Vamsi K. Yadavalli [4,*]

[1] H.E.J. Research Institute, University of Karachi, 75270, Pakistan; kanwalmuhammadiqbal43@gmail.com (K.M.I.); raza.shah@iccs.edu (M.R.S.)

[2] Department of Physics, Virginia Commonwealth University, Richmond, VA 23284, USA; mfbertino@vcu.edu

[3] Department of Forensic Science, Virginia Commonwealth University, Richmond, VA 23284, USA; cehrhardt@vcu.edu

[4] Department of Chemical and Life Science Engineering, Virginia Commonwealth University, Richmond, VA 23284, USA

* Correspondence: vyadavalli@vcu.edu; Tel.: +1-804-828-0587

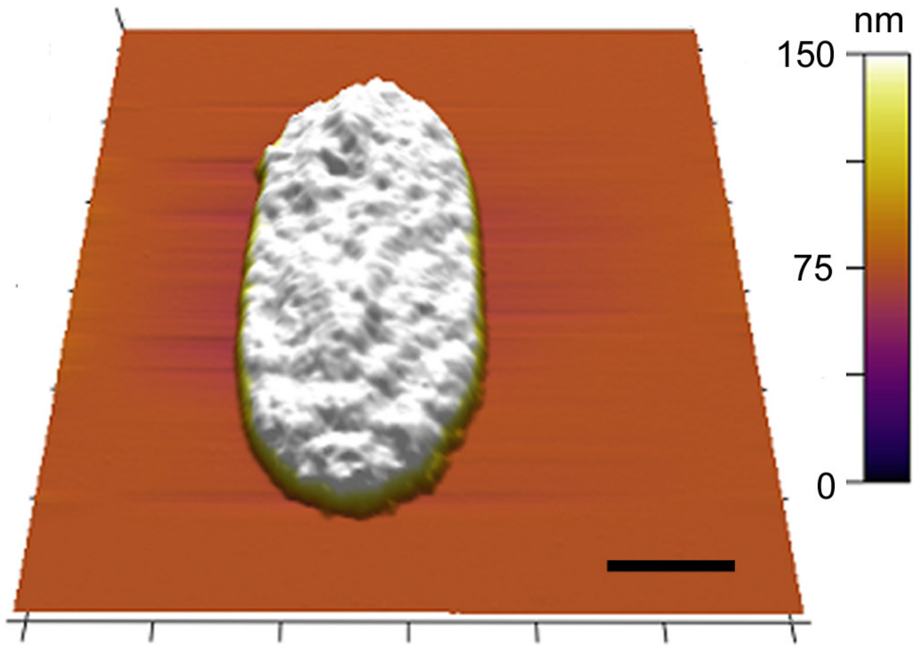

Figure S1: Atomic Force Microscope (AFM) image of *Yersinia pestis* cultured in SESOM C media. This experiment was used as a control to investigate the effect of soil on *Y. pestis* (in the experiments outlined in the manuscript, *Y. pestis* was cultured in soil using the 3D printed culture chambers). Since soil culture could be performed in the absence of the chamber, the soil extract (SESOM) was used to simulate the soil. Scale bar = 500 nm.

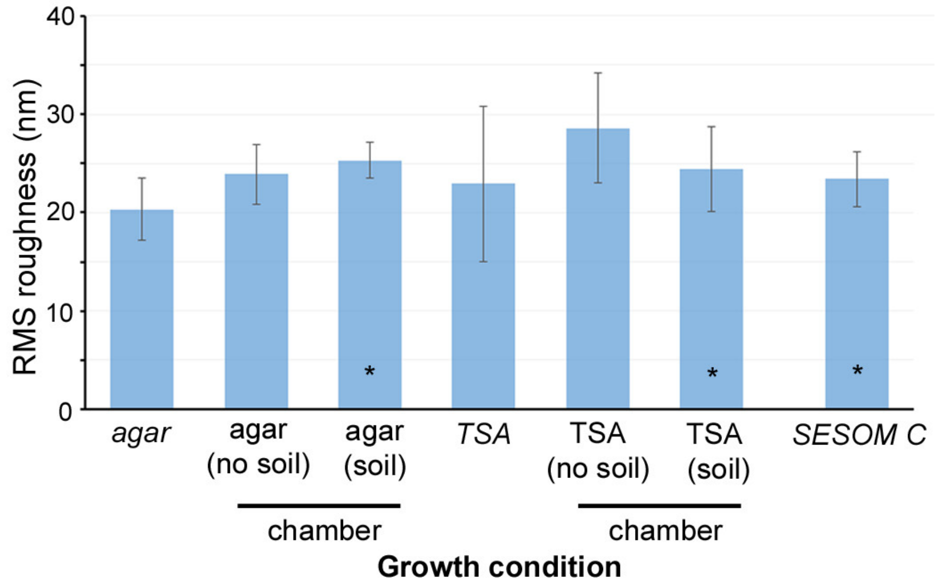

Figure S2: Cell surface root mean square (RMS) roughness in nm. Despite the different conditions, the cells present the same nanoscale morphology with no specific trend or change in roughness in different conditions. The conditions represented with an asterisk (*) are soil or soil-like conditions, whereas those in italics are cells cultured in regular plates. In contrast the chamber represents the cells cultured in the 3D printed cell culture chambers. These results further illustrate that the chamber itself did not affect the cells in any way.

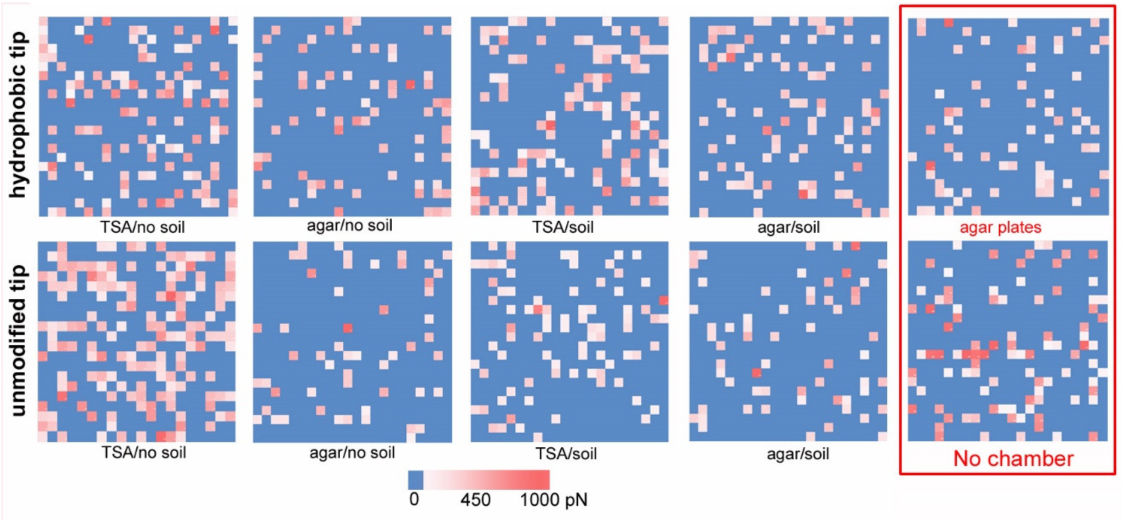

Figure S3: Surface hydrophobicity mapping of the *Yersinia pestis* cell surfaces grown under different conditions in the 3D printed culture chamber. The surfaces of the cells were probed with an unmodified AFM tip (control, bottom row) and a hydrophobic AFM tip (pendant methyl (–CH_3) groups) (top row). The changes from the two cantilevers show that the forces recorded are indeed specific to hydrophobic groups. The column on the right (red box) represents the cell culture on agar plates (no chamber) and was used as a control.

The maps are color coded as blue = no interaction with tip, white to orange = increasing interaction force with tip (ranging from 150 pN to 1 nN). The points are ~50 nm apart, thereby each square presents a profile of a 1 μm^2 area of the cell. The number of non-blue areas are representative of the points of interaction (hydrophobic groups) on the cell surface.

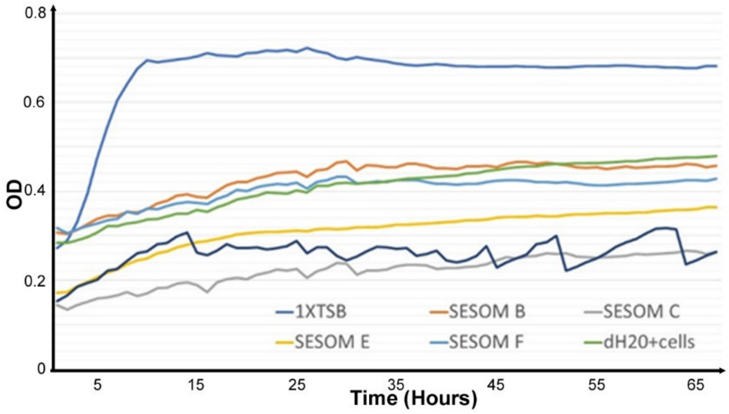

Figure S4. Growth curve comparison of *Y.pestis* KIM in TSB and SESOM media formulations.

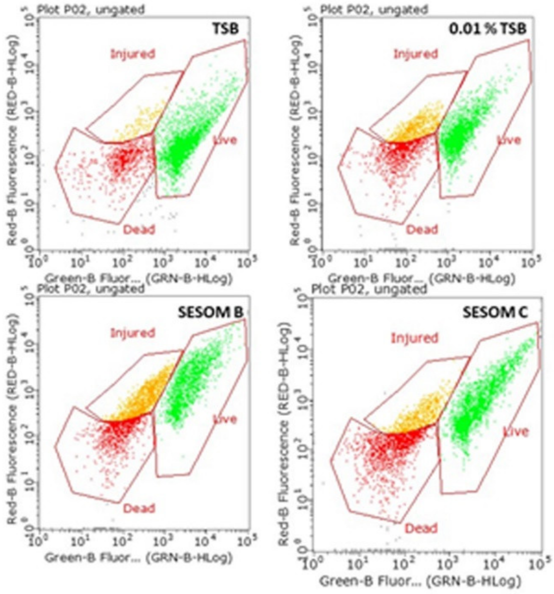

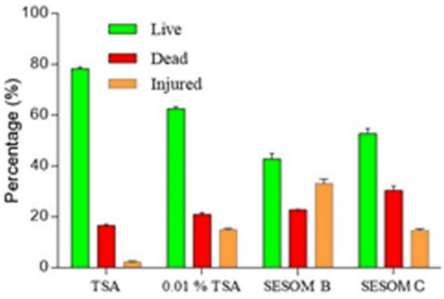

	Live (%)		Dead (%)		Injured (%)	
	Mean	SD	Mean	SD	Mean	SD
TSA	78.34	0.65	16.71	0.41	2.40	0.23
0.01% TSA	62.59	0.88	21.07	0.74	15.04	0.48
SESOM B	42.85	2.22	22.87	0.18	33.20	1.72
SESOM C	52.86	1.97	30.47	1.71	14.81	0.59

Figure S5. Viability Analysis of *Y.pestis* across TSA and SESOM Agar formulations.

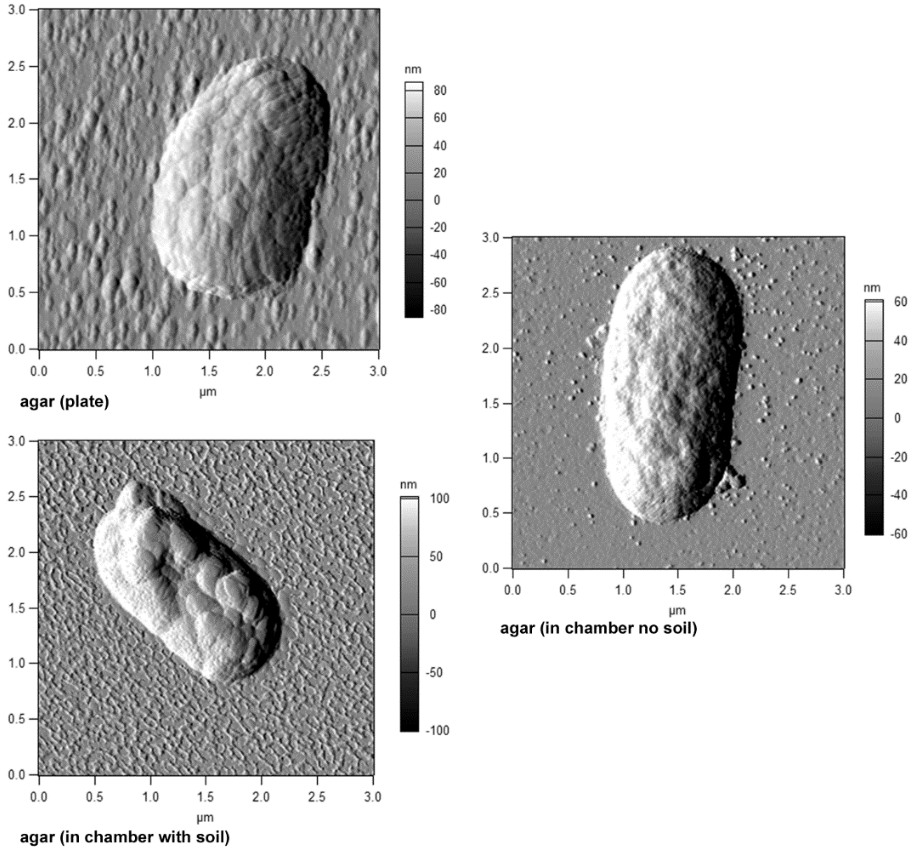

Figure S6 – Raw data showing high resolution AFM images of *Yersinia* cells in agar under the various conditions reported.

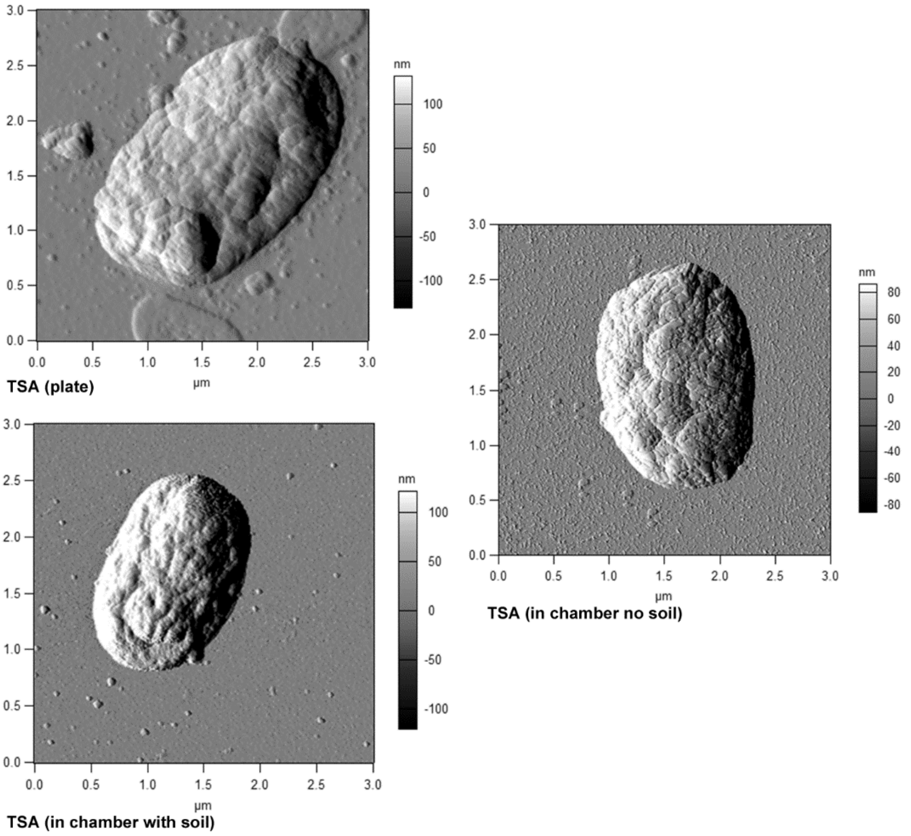

Figure S7 – Raw data showing high resolution AFM images of *Yersinia* cells in TSA under the various conditions reported.

Condition	Replicate[3]	CFU
Agar – no soil [2]	Plug 1	$3.2 \pm 0.1 \times 10^6$
	Plug 2	$2.9 \pm 0.1 \times 10^6$
Chamber – no soil	Plug 1	$1.9 \pm 1 \times 10^6$
	Plug 2	$1.0 \pm 1 \times 10^7$
Chamber - soil	Plug 1	$1.4 \pm 0.6 \times 10^7$
	Plug 2	$2.8 \pm 0.1 \times 10^7$

Table S1: Growth Chamber Viability Assay[1]

[1]CFUs provided for each culturing condition. Starting CFU inoculated on each agar plug ~2.5×10^6.

[2]Agar substrate incubated without chamber

[3]Replicates represent individual agar plugs within a single growth chamber. Two agar plugs were inoculated with *Y.pestis* KIM for each chamber. The third plug was not inoculated as a negative control to monitor for contamination from organisms outside the chamber.

Condition	% of interactions
**Agar – no soil**	13.8%
**Agar – soil**	17.8 %
**TSA – no soil**	24.4 %
**TSA – soil**	26.3 %
**Agar – no soil (no chamber)**	14.3%

Table S2: % of interactions of a hydrophobic AFM cantilever (functionalized with $–CH_3$ groups) indicative of the extent of cell surface hydrophobicity. This data is calculated from the force map representation of Figure 4.
